# Screening for genes, miRNAs and transcription factors of adipogenic differentiation and dedifferentiation of mesenchymal stem cells

**DOI:** 10.1186/s13018-023-03514-0

**Published:** 2023-01-17

**Authors:** Yi Ou-yang, Miao-miao Dai

**Affiliations:** 1 Department of Traumatic Joint Surgery, Shunde Hospital, Southern Medical University (The First People’s Hospital of Shunde, Foshan), No.1 Jiazi Road, Lunjiao, Shunde District, Foshan City, Guangdong Province China; 2 Department of Ophthalmology, Shunde Hospital, Southern Medical University (The First People’s Hospital of Shunde, Foshan), No.1 Jiazi Road, Lunjiao, Shunde District, Foshan City, Guangdong Province China

**Keywords:** miRNAs, Transcription factors, Mesenchymal stem cells, Adipogenesis, Dedifferentiation

## Abstract

**Background:**

The purpose of present study was to reveal the molecular mechanisms responsible for both adipogenic differentiation and dedifferentiation of mesenchymal stem cells (MSCs).

**Methods:**

Microarray data GSE36923 were obtained from the Gene Expression Omnibus database. Differentially expressed genes (DEGs) between adipogenically differentiated cells vs undifferentiated bone marrow-derived MSCs, adipogenically differentiated cells vs dedifferentiated cells samples at day 7 and adipogenically differentiated cells vs dedifferentiated cells samples at day 35 were screened, and overlapped DEGs across the three groups were analyzed. The underlying functions of the upregulated and downregulated DEGs were investigated by Gene ontology enrichment and Kyoto Encyclopedia of Genes and Genomes pathway analysis. The protein–protein interaction network was constructed, and hub genes were obtained subsequently. Hub genes were verified with GSE113253 dataset, and then miRNA-gene network and TF-gene network were constructed.

**Results:**

A total of 284 upregulated DEGs and 376 downregulated DEGs overlapped across the three groups. PPAR signaling pathway, AMPK signaling pathway, insulin signaling pathway, carbon metabolism, pyruvate metabolism, fatty acid metabolism, regulation of lipolysis in adipocytes, biosynthesis of amino acids, citrate cycle (TCA cycle) and 2-Oxocarboxylic acid metabolism were the top 10 pathways involving in the upregulated DEGs, and graft-versus-host disease, allograft rejection, viral myocarditis, cell adhesion molecules, phagosome, type I diabetes mellitus, antigen processing and presentation, autoimmune thyroid disease, intestinal immune network for IgA production and rheumatoid arthritis were the top 10 pathways in downregulated DEGs. After validation, the 8 hub genes were IL6, PPARG, CCL2, FASN, CEBPA, ADIPOQ, FABP4 and LIPE. Ten key miRNAs were hsa-mir-27a-3p, hsa-mir-182-5p, hsa-mir-7-5p, hsa-mir-16-5p, hsa-mir-1-3p, hsa-mir-155-5p, hsa-mir-21-3p, hsa-mir-34a-5p, hsa-mir-27a-5p and hsa-mir-30c-5p, and 10 key TFs were TFDP1, GTF2A2, ZNF584, NRF1, ZNF512, NFRKB, CEBPG, KLF16, GLIS2 and MXD4.

**Conclusion:**

Our study constructed miRNA-gene network and TF-gene network involved in both adipogenic differentiation and dedifferentiation of MSCs, contributing to enhancing the efficiency of MSCs transplantation in soft tissue defect repair and developing more potent remedies for adipogenesis-related skeletal disorders.

**Supplementary Information:**

The online version contains supplementary material available at 10.1186/s13018-023-03514-0.

## Background

Mesenchymal stem cells (MSCs) are multipotent stromal cells that could differentiate into adipogenic, osteogenic, chondrogenic and other lineages[1]. MSCs derive from many tissues, such as bone marrow, umbilical cord, placenta, skin and fat[2]. MSCs has attracted increasing interest in regenerative medicine for easy isolation, rapid proliferation, low immunogenicity and multilineage differentiation potential[3]. Adipogenic differentiation is an important direction of MSCs differentiation for that it plays an important role in soft tissue defect repair in traumatology[4]. Moreover, excessive adipogenic differentiation of MSCs is related to skeletal diseases such as osteoporosis and osteonecrosis[5, 6]. Therefore, regulation of adipogenic differentiation of MSCs is of great importance for clinical work in orthopedics.

Adipogenic differentiation has been regarded as an irreversible terminal process for a long time[7]. However, Sugihara et al.[8, 9] first found that mature adipocytes could dedifferentiated into fibroblast-like cells using ceiling culture technology. These fibroblast-like cells, termed dedifferentiated fat cells, possess stem cell properties, and can re-differentiate to multiple mesenchymal cell lineages[10]. Adipocyte differentiation and dedifferentiation are two opposite processes, however, the mechanisms responsible for adipocyte dedifferentiation remain largely unknown. Generally, genes and pathways that are required for adipogenesis are also essential for dedifferentiation[11]. The mechanism involved in both adipogenic differentiation and dedifferentiation may become a key to regulating MSC adipogenesis.

Adipogenic differentiation and dedifferentiation may involve signaling pathways including PPARγ pathway[12, 13], Wnt/β-catenin pathway[14, 15], transforming growth factor-β pathway[16, 17], and Notch signaling pathway[13, 18]. Moreover, microRNAs such as miR-377-3p[19] and miR-431[20] and transcription factors (TFs) like FOXN1[21] and TEAD4[22] may also be involved in the adipogenic differentiation. The role of microRNAs and TFs in adipocyte dedifferentiation, however, remains largely unknown. Therefore, molecular mechanism of MSCs adipogenic differentiation and dedifferentiation is complicated, and it is necessary to elucidate that for further application.

To explore the molecular mechanisms responsible for the adipogenic differentiation and dedifferentiation of MSCs, we screened the differentially expressed genes (DEGs) of microarray data GSE36923. Subsequently, comprehensive bioinformatics methods were used to analyze the functions and potential pathways of DEGs. In addition, protein–protein interaction (PPI) network was performed to elucidate the underlying molecular mechanisms. Hub genes were calculated and then validated using dataset GSE113253. Furthermore, TFs and miRNAs targeting the hub genes were predicted, and miRNA-gene network and TF-gene network were constructed.

## Methods

### Microarray data

The gene expression profile of GSE36923 was downloaded from Gene Expression Omnibus database (GEO, http://www.ncbi.nlm.nih.gov/geo/). GSE36923 was based on the Affymetrix GPL570 platform (Affymetrix Human Genome U133 Plus 2.0 Array, Santa Clara, CA, USA). The GSE36923 dataset contained 12 samples, including 3 undifferentiated bone marrow-derived MSCs (BMSCs) samples, 3 adipogenically differentiated cells samples at d 15, 3 dedifferentiated cells samples at d 7 and 3 dedifferentiated cells samples at d 35. MSCs features, adipogenic differentiation and dedifferentiation method were showed as Ullah, M et al. described[23].

### Screening of differentially expressed genes

NetworkAnalyst (https://www.networkanalyst.ca/NetworkAnalyst/), an online platform of transcriptome profiling, network analysis, and meta-analysis for gene expression data[24], was used to screen DEGs from the gene expression data. Data was normalized by variance stabilizing normalization followed by quantile normalization. Box plots for expression data were generated. DEGs (adipogenically differentiated cells vs undifferentiated MSCs, adipogenically differentiated cells vs dedifferentiated cells samples at d 7 and adipogenically differentiated cells vs dedifferentiated cells samples at d 35) were screened with the criteria of adjust P value < 0.05 and |logFC (fold change)|≥ 1. Overlapped DEGs across the three groups were analyzed and visualized using R software (version 3.6.3; https://www.r-project.org/) and ggplot2 package (version 3.3.3). Heatmap indicating all overlaps between up- and downregulated genes for all comparisons was visualized using R software (version 3.6.3; https://www.r-project.org/) and pheatmap package (version 1.0.12).

### Gene ontology enrichment and KEGG pathway analysis

Gene ontology (GO) enrichment and Kyoto Encyclopedia of Genes and Genomes (KEGG) pathway analysis of upregulated and downregulated DEGs were performed using R software (version 3.6.3; https://www.r-project.org/) and clusterProfiler packages (version 3.14.3)[25]. P value < 0.05 was considered to indicate a statistical significance.

### PPI network construction and hub genes selection

The PPI network was constructed using The Search Tool for the Retrieval of Interacting Genes/Proteins (http://string-db.org)[26] with the threshold of minimum required interaction score > 0.4. Network and node file were generated and further analyzed using Cytoscape software (version 3.8.2; www.cytoscape.org). Cytoscape's plug-in cytoHubba was used to identify top 100 key genes by degree algorithm, and the PPI network of top 100 key genes was constructed.

### Hub genes validation

Top ten hub genes were further validated in GSE113253, downloaded from the GEO database. The dataset was based on the platform of GPL18460 (Illumina HiSeq 1500), in which 3 undifferentiated BMSCs and 3 adipogenically differentiated cells samples at d 14 were selected. MSCs features and adipogenic differentiation method were showed as Rauch et al. described [27]. DEGs were identified with Student’s *t*-test. P value of < 0.05 was considered statistically significant.

### MiRNA-target gene network and TF-target gene network construction

After validation, eight genes (IL6, PPARG, CCL2, FASN, CEBPA, ADIPOQ, FABP4, LIPE) were selected as target genes. MiRNA-target gene network and TF-target gene network were analyzed using TarBase (version 8.0; https://dianalab.e-ce.uth.gr/html/diana/web/index.php?r=tarbasev8) and ENCODE software package (http://cistrome.org/BETA/) in NetworkAnalyst, and were visualized by employing Cytoscape software. Top 10 key miRNAs and TFs were identified with cytoHubba by degree algorithm.

## Results

### Identification of DEGs during MSC adipogenesis and dedifferentiation

**Fig. 1:**
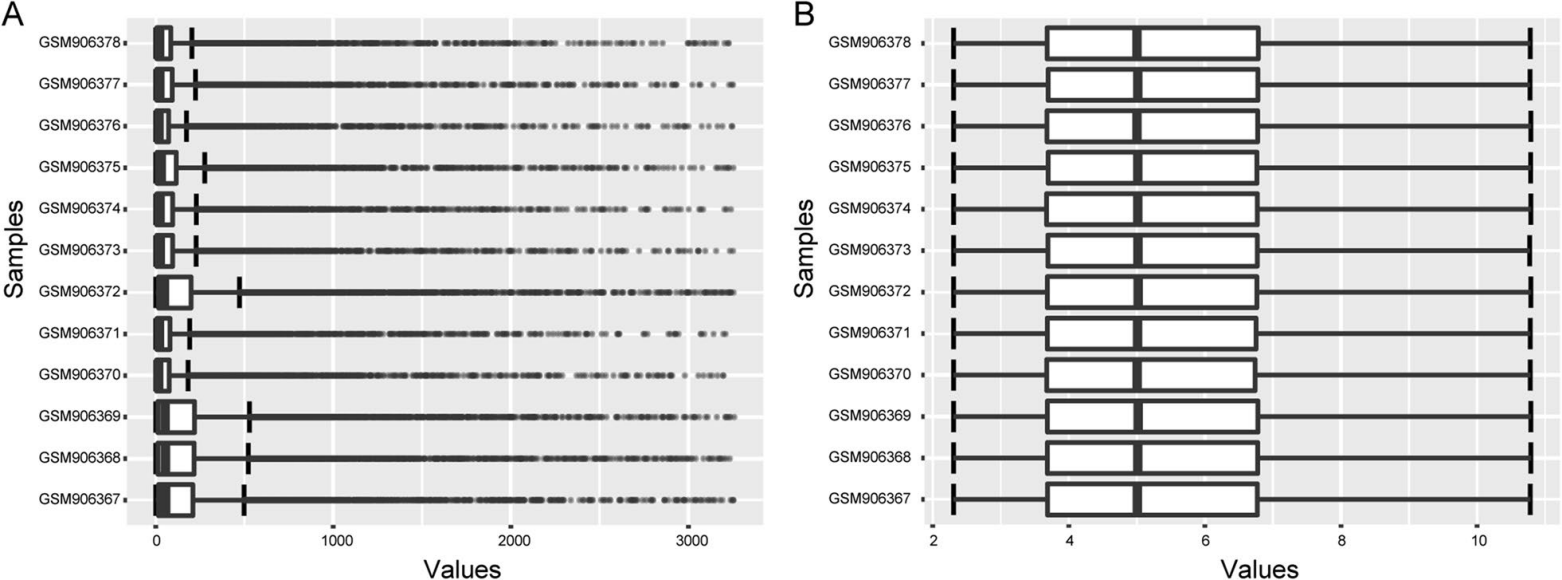
Box plots before normalization (**A**) and after normalization (**B**). The vertical axis is the name of samples while the horizontal axis stands for the values of expression. The black line stands for the median of data and represents the normalization degree. After normalization, black line in each group was almost collinear, which indicates an excellent degree of normalization

**Fig. 2:**
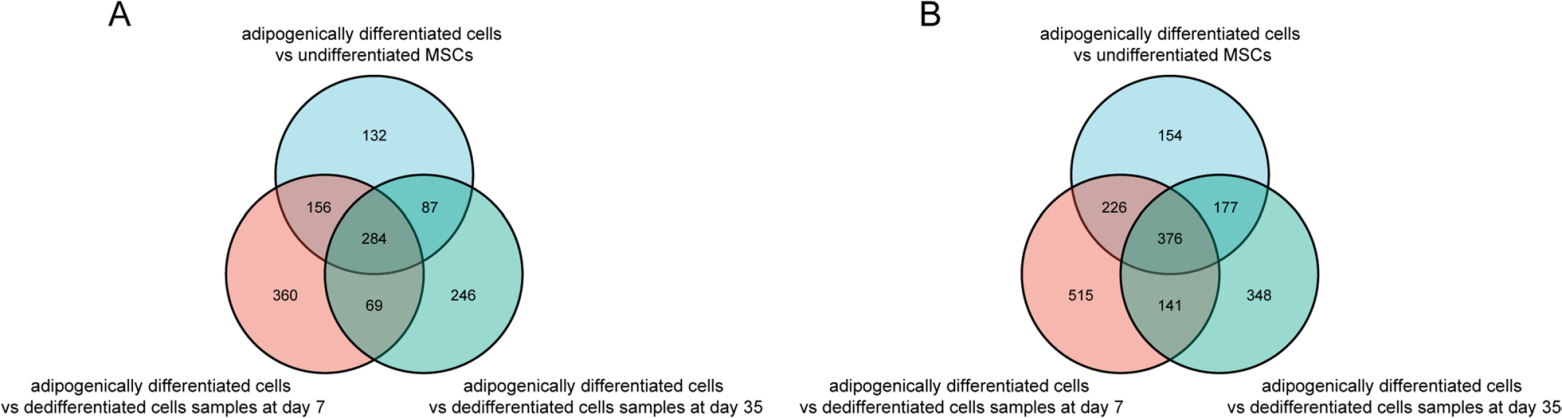
The Venn diagram of overlapped upregulated (**A**) and downregulated (**B**) differentially expressed genes of adipogenically differentiated cells vs undifferentiated MSCs, adipogenically differentiated cells vs dedifferentiated cells samples at d 7 and adipogenically differentiated cells vs dedifferentiated cells samples at d 35

**Fig. 3:**
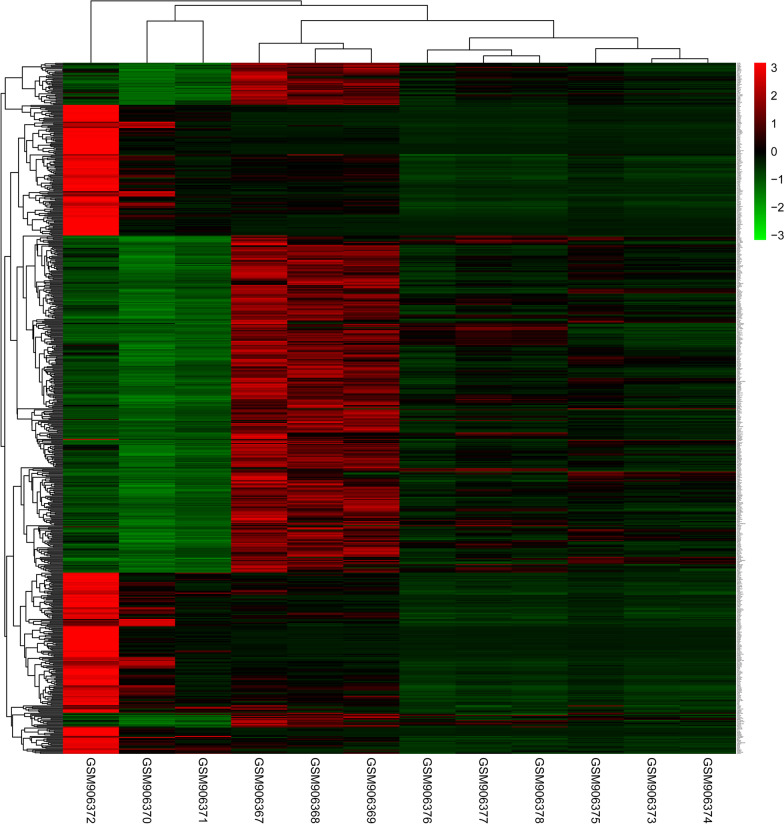
Heatmap indicating all overlap genes. Red represents high expression, and the deeper the red color, a higher expression value. Green represents low expression, and a deeper green color, a lower the expression value

After normalization (Fig. 1), 659, 869 and 686 DEGs were upregulated and 933, 1258 and 1042 DEGs were downregulated in adipogenically differentiated cells vs undifferentiated MSCs, adipogenically differentiated cells vs dedifferentiated cells samples at d 7 and adipogenically differentiated cells vs dedifferentiated cells samples at d 35, respectively. A total of 284 upregulated DEGs and 376 downregulated DEGs overlapped across the three groups (Fig. 2) and were shown in the heatmap (Fig. 3).

### GO enrichment and KEGG pathway analysis

**Fig. 4:**
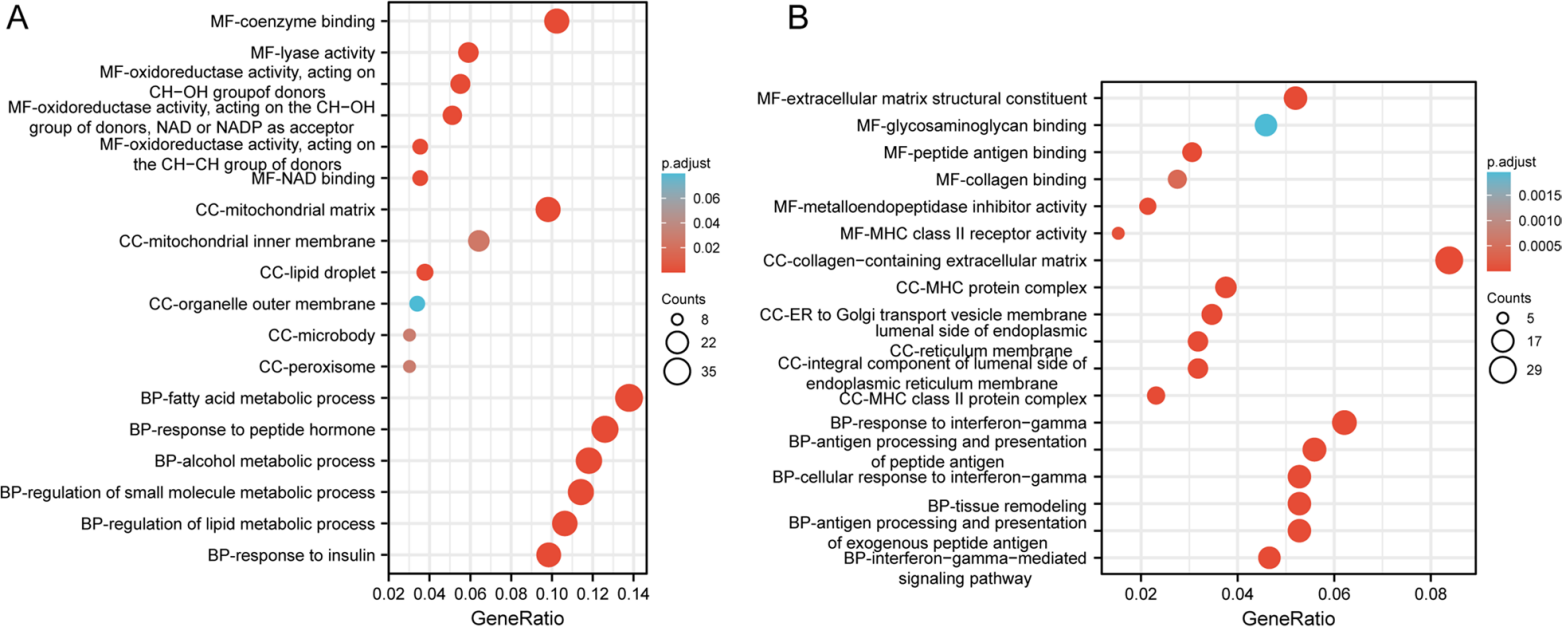
Gene Ontology (GO) enrichment results of overlapped upregulated (**A**) and downregulated (**B**) differentially expressed genes (DEGs). Bubble charts show GO enrichment significance items of DEGs in three functional groups: molecular function (MF), cellular component (CC) and biological process (BP). The x-axis label represents the gene ratio, and the y-axis label represents GO terms. The size of circle stands for gene count and the color of circle stands for adjusted p value

**Fig. 5:**
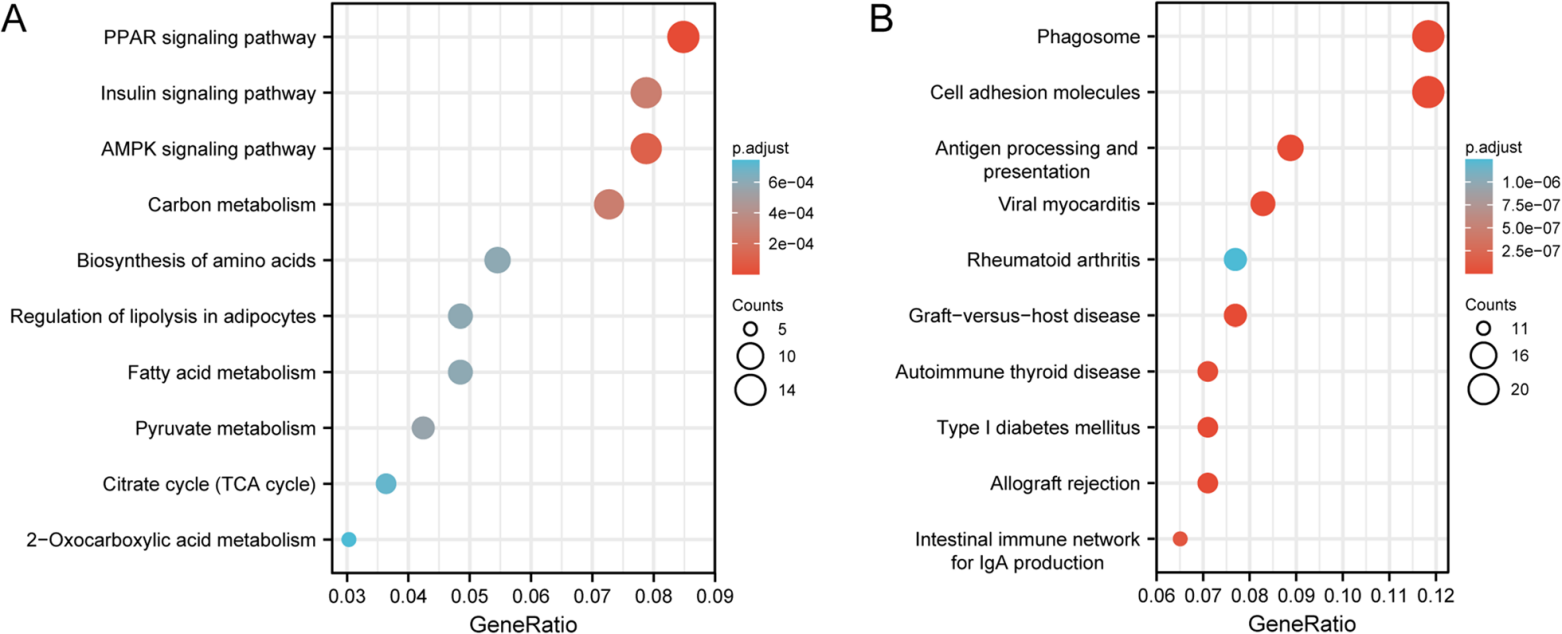
Kyoto Encyclopaedia of Genes and Genomes (KEGG) pathway analysis of overlapped upregulated (**A**) and downregulated (**B**) differentially expressed genes (DEGs). Bubble charts show enrichment of DEGs in signaling pathways. The x-axis label represents the gene ratio, and the y-axis label represents pathway. The size and color of circle stand for gene count in pathway and adjusted p value, respectively

In order to elucidate the functions of overlapped DEGs, upregulated and downregulated genes were further analyzed for functional enrichment, respectively. With regard to the molecular function, upregulated DEGs (Fig. 4A) mainly showed enrichment in the coenzyme binding, oxidoreductase activity, acting on CH-OH group of donors, oxidoreductase activity, acting on the CH-OH group of donors, NAD or NADP as acceptor, lyase activity, NAD binding and oxidoreductase activity, acting on the CH-CH group of donors, and downregulated DEGs (Fig. 4B) mainly showed enrichment in the peptide antigen binding, metalloendopeptidase inhibitor activity, extracellular matrix structural constituent, MHC class II receptor activity, collagen binding and glycosaminoglycan binding. For the cellular component ontology (Fig. 4A), commonly enriched categories were associated with mitochondrial matrix, lipid droplet, mitochondrial inner membrane, peroxisome, microbody and organelle outer membrane in upregulated DEGs, and were associated with MHC protein complex, integral component of lumenal side of endoplasmic reticulum membrane, lumenal side of endoplasmic reticulum membrane, MHC class II protein complex, collagen-containing extracellular matrix and ER to Golgi transport vesicle membrane in downregulated DEGs (Fig. 4B). For the biological process (Fig. 5A), fatty acid metabolic process, alcohol metabolic process, response to peptide hormone, response to insulin, regulation of small molecule metabolic process and regulation of lipid metabolic process were the commonly enriched categories in upregulated DEGs, and interferon-gamma-mediated signaling pathway, response to interferon-gamma, antigen processing and presentation of peptide antigen, antigen processing and presentation of exogenous peptide antigen, tissue remodeling and cellular response to interferon-gamma in downregulated DEGs (Fig. 4B). PPAR signaling pathway, AMPK signaling pathway, insulin signaling pathway, carbon metabolism, pyruvate metabolism, fatty acid metabolism, regulation of lipolysis in adipocytes, biosynthesis of amino acids, citrate cycle (TCA cycle) and 2-Oxocarboxylic acid metabolism were the top 10 pathways involving in the upregulated DEGs (Fig. 5A), and graft-versus-host disease, allograft rejection, viral myocarditis, cell adhesion molecules, phagosome, type I diabetes mellitus, antigen processing and presentation, autoimmune thyroid disease, intestinal immune network for IgA production and rheumatoid arthritis in downregulated DEGs (Fig. 5B).

### Protein–protein interaction network

**Fig. 6:**
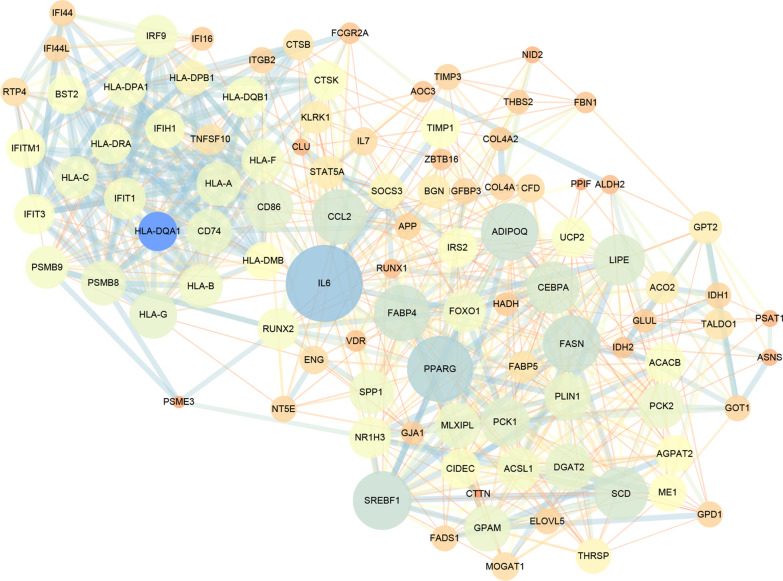
Protein–protein interaction network. The circle represents genes and the line indicates the interactions among genes. A thicker line stands for a higher edge confidence, and a larger node size stands for a higher degree

The protein–protein interaction network of top 100 key genes is shown in Fig. 6. The top 10 high-degree hub nodes were IL6, PPARG, CCL2, FASN, SREBF1, SCD, CEBPA, ADIPOQ, FABP4 and LIPE.

### Validation of hub genes expression

**Fig. 7:**
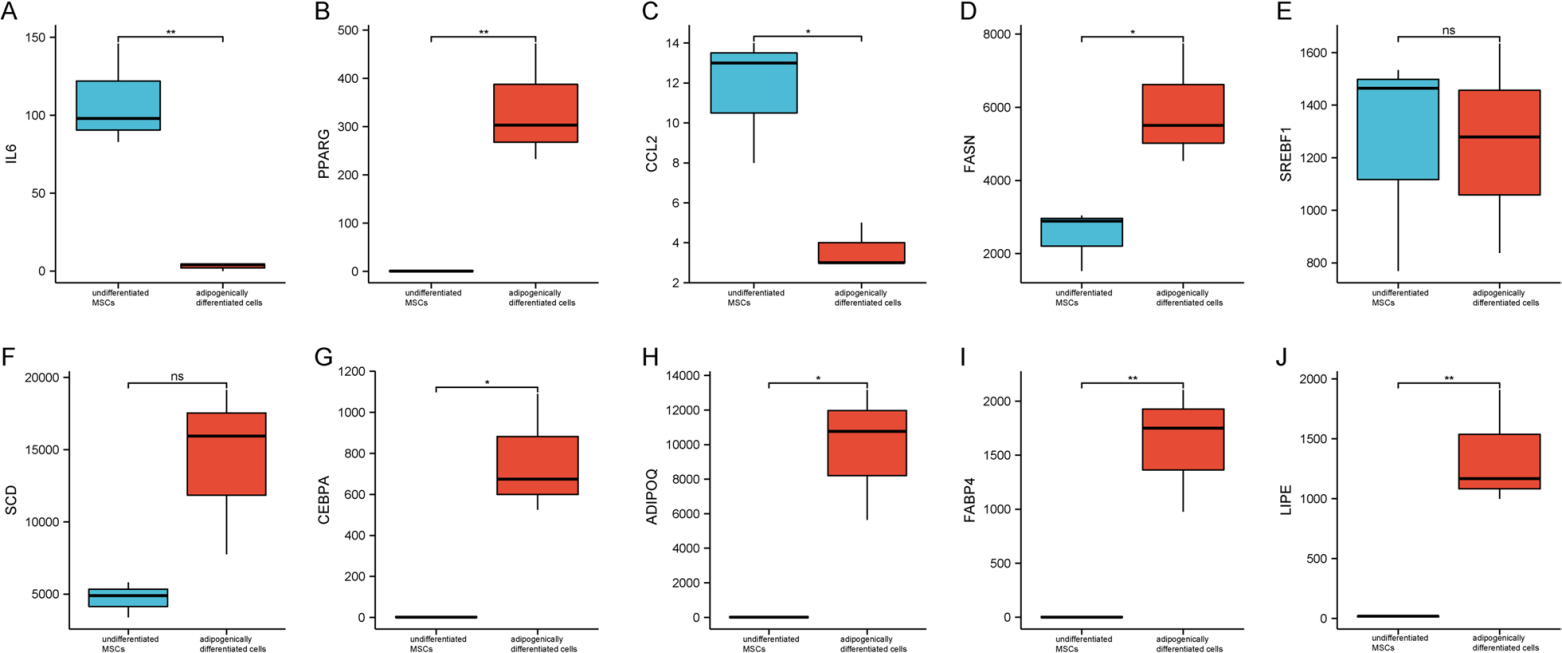
Validation of top 10 hub genes in dataset GSE113253. Expression levels of PPARG (**B**), FASN (**D**), CEBPA (**G**), ADIPOQ (**H**), FABP4 (**I**) and LIPE (**J**) were significantly upregulated in adipogenically differentiated cells. Expression levels of IL6 (**A**) and CCL2 (**C**) were significantly downregulated in adipogenically differentiated cells. There were no statistically significant differences in the expression levels of SREBF1 (**E**) and SCD (**F**) between undifferentiated MSCs and adipogenically differentiated cells

Ten hub genes were validated using the GSE113253 dataset. For upregulated hub genes, compared to undifferentiated MSCs, the expressions of PPARG, FASN, CEBPA, ADIPOQ, FABP4 and LIPE were consistently increased in adipogenically differentiated cells (Fig. 7B, D, G–J). For downregulated hub genes, the expression of IL6 and CCL2 also decreased during adipogenesis (Fig. 7A, C). Results demonstrated that IL6, PPARG, CCL2, FASN, CEBPA, ADIPOQ, FABP4 and LIPE were pivotal genes involved in adipogenic differentiation and dedifferentiation of MSCs (Table 1).

**Table 1:** Hub genes and their descriptions

Gene symbol	Description	Degree score
IL6	interleukin 6	174
PPARG	peroxisome proliferator activated receptor gamma	108
CCL2	C–C motif chemokine ligand 2	94
FASN	fatty acid synthase	82
CEBPA	CCAAT enhancer binding protein alpha	76
ADIPOQ	adiponectin, C1Q and collagen domain containing	74
FABP4	fatty acid binding protein 4	72
LIPE	lipase E, hormone sensitive type	70

### MiRNA-target gene interaction network construction

**Fig. 8:**
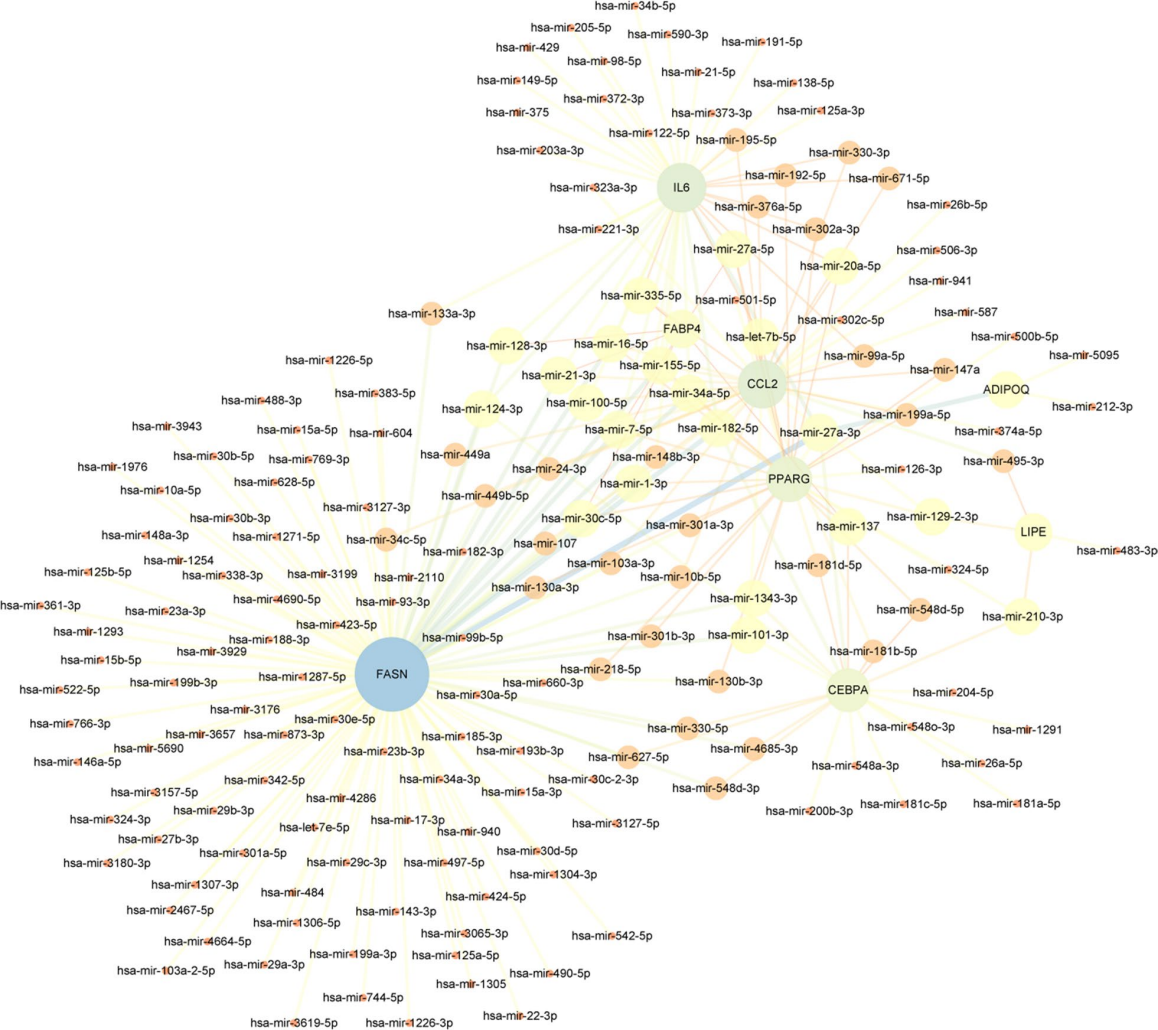
The miRNA-target gene interaction network. The circle represents genes and the line indicates the interactions among genes. A thicker line stands for a higher edge betweenness, and a larger node size stands for a higher degree

174 miRNAs targeting 8 target hub genes were obtained in NetworkAnalyst using TarBase (version 8.0), and an additional table file shows this in more detail [see Additional file 1]. The miRNA-target gene interaction network was constructed with cytoscape (Fig. 8). According to the nodes’ degree value, top 10 key miRNAs were hsa-mir-27a-3p, hsa-mir-182-5p, hsa-mir-7-5p, hsa-mir-16-5p, hsa-mir-1-3p, hsa-mir-155-5p, hsa-mir-21-3p, hsa-mir-34a-5p, hsa-mir-27a-5p and hsa-mir-30c-5p.

### TF-target gene interaction network construction

**Fig. 9:**
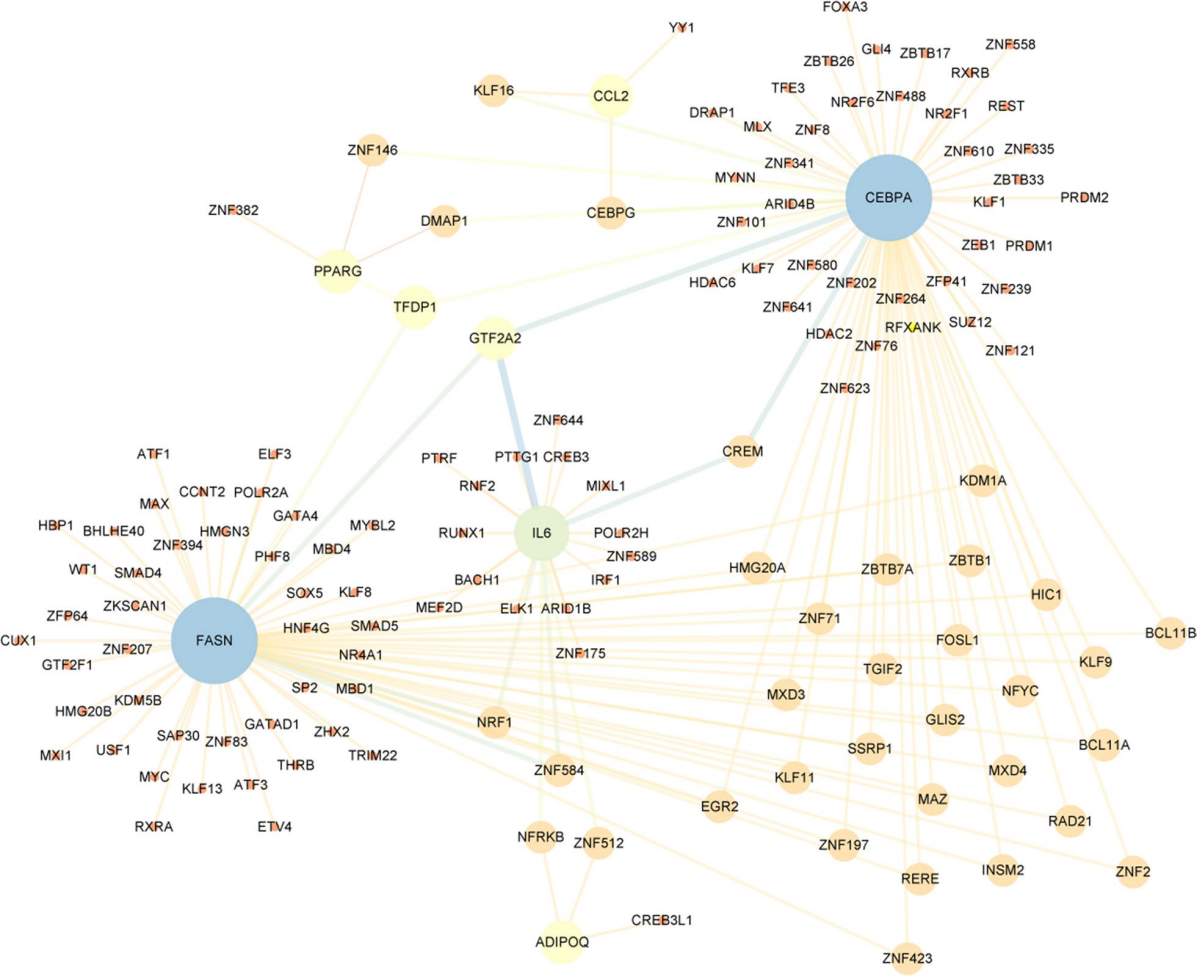
The TF-target gene interaction network. The circle represents genes and the line indicates the interactions among genes. A thicker line stands for a higher edge betweenness, and a larger node size stands for a higher degree

135 TFs targeting 8 hub genes were obtained in NetworkAnalyst using ENCODE software package, and an additional table file shows this in more detail [see Additional file 2]. The TF-target gene interaction network was also constructed with cytoscape (Fig. 9). According to the nodes’ degree value, top 10 key TFs were TFDP1, GTF2A2, ZNF584, NRF1, ZNF512, NFRKB, CEBPG, KLF16, GLIS2 and MXD4.

## Discussion

MSCs are promising seed cells in tissue engineering. These cells are usually applied in restoring large-sized soft tissue defects resulting from trauma, musculoskeletal tumor resection and congenital defects for their adipogenetic ability [28]. However, adipocyte dedifferentiation would occur after transplantation [29]. Moreover, MSCs adipogenesis is correlated with diseases like osteoporosis and osteonecrosis. Complete elucidation of the mechanism underlying the adipogenetic differentiation and dedifferentiation could contribute to designing better seed cells based on MSCs in tissue engineering and discovering more effective treatments for skeletal diseases related to adipogenesis [11].

In this study, we analyzed DEGs of adipogenically differentiated MSCs and undifferentiated MSCs, adipogenically differentiated MSCs and dedifferentiated MSCs at d 7, and adipogenically differentiated MSCs vs dedifferentiated MSCs at d 35, respectively. Overlapped DEGs across the three groups contained 284 upregulated genes and 376 downregulated genes. GO and KEGG pathway analyses were carried out to find the interactions of DEGs. For the upregulated DEGs, pathway enrichment analysis revealed that PPAR signaling pathway was significantly enriched. PPAR-γ has been reported to be one of the important genetic factors in the MSCs adipogenic differentiation and dedifferentiation, and PPAR signaling pathway plays a role in the regulation of adipocyte differentiation and dedifferentiation [11, 30–32]. For the downregulated DEGs, pathway enrichment analysis revealed that the graft-versus-host disease (GVHD) was significantly enriched. Qi et al. [33] revealed that the adipogenic differentiation capacity was decreased in the active chronic GVHD MSCs compared with no-cGVHD MSCs. The adipogenesis of MSCs may be negatively associated with GVHD disease, which was in line with the results of the present study.

After PPI network construction and validation, we found 8 hub genes including IL6, PPARG, CCL2, FASN, CEBPA, ADIPOQ, FABP4 and LIPE. PPARG, FASN, SREBF1, SCD, CEBPA, ADIPOQ, FABP4 are key adipogenic markers, and activation expression of these genes could enhance the adipogenic differentiation of MSCs [34–38]. PPARG, CEBPA, ADIPOQ and FABP4 are involved in adipogenesis, and FASN is involved in lipogenesis. Ullah et al. [23] had confirmed that the expression of ADIPOQ and FABP4 upregulated at adipogenic differentiation d 15 compared to undifferentiated MSCs d 0, and downregulated during dedifferentiation. LIPE, a gene related to lipolysis, plays a key role in the regulation of adipose tissue deposition [39, 40]. Yi et al. [41] found that the expression of ADIPOQ, CEBPA, FABP4, FASN AND LIPE were significantly higher during adipogenesis of MSCs by the RNA-Seq technique, which is consistent with our results. Liao et al. [42] found that the expression of PPARG, CEBPA and ADIPOQ was significantly downregulated in vivo dedifferentiation of adult adipose cells. Cote et al. [43] explored the changes in gene expression profile during human mature adipocyte dedifferentiation, they also found that the expression of LIPE, ADIPOQ, CEBPA AND FABP4 decreased when human mature adipocyte dedifferentiated from d 7 to d 12. However, they used ceiling culture technique for adipocyte dedifferentiation, which is different with our study. IL6 is a known regulator of adipose homeostasis in obesity and is high secreted from adipose tissue [44, 45]. It involves in obesity-associated metabolic complications [45]. Moreover, IL6 involves in differentiation of MSCs. IL6 could enhance osteogenetic and myogenetic differentiation of MSCs [46–48]. Huang et al. [49] found that IL6 potentiated BMP-2-induced osteogenesis and adipogenesis of BMSCs by amplifying BMPR1A-mediated BMP/Smad and p38 MAPK pathways, respectively. CCL2 is a chemokine that plays an important role in inflammation and indirectly involves in the process of adipogenesis via inflammatory responses. Zhu et al. [50] revealed that CCL2 directly promoted adipogenesis of MSCs in vitro possibly by enhancing AKT phosphorylation.

Among these 10 hub miRNAs, we found that the interactions between adipogenesis and hub miRNAs hsa-mir-21-3p, hsa-mir-27a-5p and hsa-mir-30c-5p have not been reported. MiR-27-3p exerts an inhibitory effect on adipogenesis by repressing PPARγ [51, 52]. Ding et al. [53] found that circPTK2 promoted lipolysis and inhibited adipogenesis through binding to miR-182-5p. Chen et al. [54] found that CDR1as-miR-7-5p-WNT5B axis might play crucial role in adipogenic/osteogenic differentiation disorder of BMSCs from steroid-induced osteonecrosis of the femoral head patients. Xu et al. [55] found that miR-16-5p promoted adipocyte differentiation by suppressing EPT1. Gu et al. [56] found that miR-1-3p was upregulated during osteogenesis but downregulated during adipogenesis of mouse MSCs. Du et al. [57] found that hsa-miR-1-3p was one of hub miRNAs coregulated both osteogenic and adipogenic differentiation. Eseberri et al. [58] found that resveratrol and glucuronide metabolites inhibited adipogenesis through upregulating miR-155 expression. Meruvu et al. [59] found that butyl benzyl phthalate promoted adipogenesis via the miRNA-34a-5p signaling pathway.

Among these 10 hub TFs, we found that the interactions between adipogenesis and hub TFs TFDP1, GTF2A2, ZNF584, ZNF512, NFRKB and GLIS2 have not been reported. Xue et al. [60] found that long isoforms of NRF1 was a negative regulator of PPARγ expression to suppress adipogenesis. CEBPG is one of potential TFs involved in adipogenesis[61]. Jang et al.[62] found that KLF16 inhibited adipogenesis through downregulating PPARγ expression. However, Cui et al. [63] found that when the FGF1 upregulated, adipocytes accumulation accelerated, while the expression of KLF16 increased significantly at the same time. Li et al. [64] found that over-expression of MXD4 could inhibit adipogenesis of human adipose-derived stem cells.

## Conclusions

In conclusion, we have identified key genes, miRNAs and TFs involved in both adipogenic differentiation and dedifferentiation of MSCs by bioinformatical analyses and constructed the miRNA-gene network and TF-gene network. These findings could improve our understanding of adipogenic differentiation and dedifferentiation of stem cells, contributing to enhancing the efficiency of MSCs transplantation in soft tissue defect repair and developing more potent remedies for adipogenesis-related skeletal disorders.

## Supplementary Information

**Additional file 1.** miRNAs and their degree scores

**Additional file 2.** TFs and their descriptions

## Data Availability

The datasets supporting the conclusions of this article are available in the Gene Expression Omnibus repository, https://www.ncbi.nlm.nih.gov/geo/query/acc.cgi?acc=GSE36923 and https://www.ncbi.nlm.nih.gov/geo/query/acc.cgi?acc=GSE113253.

## Abbreviations

MSCs: Mesenchymal stem cells
DEGs: Differentially expressed genes
PPI: Protein–protein interaction
BMSCs: Bone marrow-derived mesenchymal stem cells
GO: Gene ontology
KEGG: Kyoto encyclopedia of genes and genomes
GVHD: Graft-versus-host disease
